# Oxidative Stress in Myopia

**DOI:** 10.1155/2015/750637

**Published:** 2015-04-01

**Authors:** Bosch-Morell Francisco, Mérida Salvador, Navea Amparo

**Affiliations:** ^1^Instituto de Ciencias Biomédicas, Universidad CEU Cardenal Herrera, Avenida del Seminario s/n, Moncada, 46313 Valencia, Spain; ^2^FISABIO, Oftalmología Médica, Bifurcación Pío Baroja-general Aviles, S/N, 46015 Valencia, Spain

## Abstract

Myopia affected approximately 1.6 billion people worldwide in 2000, and it is expected to increase to 2.5 billion by 2020. Although optical problems can be corrected by optics or surgical procedures, normal myopia and high myopia are still an unsolved medical problem. They frequently predispose people who have them to suffer from other eye pathologies: retinal detachment, glaucoma, macular hemorrhage, cataracts, and so on being one of the main causes of visual deterioration and blindness. Genetic and environmental factors have been associated with myopia. Nevertheless, lack of knowledge in the underlying physiopathological molecular mechanisms has not permitted an adequate diagnosis, prevention, or treatment to be found. Nowadays several pieces of evidence indicate that oxidative stress may help explain the altered regulatory pathways in myopia and the appearance of associated eye diseases. On the one hand, oxidative damage associated with hypoxia myopic can alter the neuromodulation that nitric oxide and dopamine have in eye growth. On the other hand, radical superoxide or peroxynitrite production damage retina, vitreous, lens, and so on contributing to the appearance of retinopathies, retinal detachment, cataracts and so on. The objective of this review is to suggest that oxidative stress is one of the key pieces that can help solve this complex eye problem.

## 1. Introduction

Myopia is a health problem found in many parts of the world and myopia prevalence has been particularly studied in East Asia. Initially, myopia has been especially associated with the Chinese population for its ethnic genetic differences, and not only for the high levels in this country, but also because of its high prevalences reported in several eastern countries and in Chinese adults: 38.7% in Singapore [1] or 40% in Hong Kong [2]. Yet if it is compared with some population studies conducted in Asian countries, the values do not differ that much in similar age groups: 34.7% in South Korea [3], 34.6% in India [4] or 41.8% in Japan [5]. However it is a mistaken view that myopia is a problem only in Asia. A recent study done in the United States obtained not very different values for Chinese and white participants (37.2% and 31.0%), but clearly much lower values for Hispanic (14.2%) and coloured (21.5%) participants. In Western Europe, the described prevalence was 26.6% [6]. Other studies have stressed the importance of differentiating between urban or rural life as differences were found between these two population types in the same country and in migratory populations [7]. What came over quite clearly was that it is a serious problem worldwide, and even more so when we consider that prevalence of myopia has increased in recent decades and that it affects 10–20% of those completing secondary school education [8, 9] in some parts of the world. What all this seems to indicate is that myopia levels will continue to rise in forthcoming years and will reach 2.5 billion by the year 2020 [6].

One key question behind the myopia concept actually lies in two clearly different problems. On the one hand, it is an optical problem poor focussing due to a mismatch between eyeball axial length and the lenses composing it (cornea and crystalline). On the other hand, it is still an unsolved medical problem that predisposes a person who has it to suffer other eye pathologies more frequently: retinal detachment, glaucoma, macular haemorrhaging, cataracts, and so forth [10]. Such pathologies alone may represent almost 40% of the surgical pathology of a specialised retina service, which implies high-cost healthcare resources. It is a serious mistake to ignore this situation after correcting an optical problem by surgical procedures [11]. The most suitable way to likely deal with both problems is that basically two types of myopia exist, each with a different prognosis, although there are no well-defined limits. Traditionally, normal myopia (NM) is slight or moderate and is associated with less than 6 negative ametropic dioptres or axial eyeball length under 26 mm. Any higher value is considered high myopia (HM), also known as magna, degenerative, progressive or malign myopia. HM is characterised by an eyeball length that uninterruptedly becomes longer during one's lifetime. This produces progressive atrophy of eye tissues and leads to blindness in a large percentage of the affected population. Therefore, HM is not only NM with more diopters but is also a serious unsolved disease.

HM prevalence in people aged over 40 is 4–8% in the USA, west Europe, Australia and Asia, but it also seems to be somewhat higher in Asian populations [6, 12] and, once again, myopia appears to be on in the increase worldwide [13–15]. Two factors make slowing down this “silent epidemic” difficult: lack of adequate treatment, as mentioned later on, and the fact that the only differentiating parameter between NM and HM is having more than −6 D or an eyeball axial length longer than 26 mm, with practically irreversible damage to the retina. Identifying a molecular pattern is, therefore, essential to make an early diagnosis and/or to follow-up this disease and set new perspectives and therapeutic targets, and always in the interest of obtaining more efficient, sustainable healthcare for these patients. It is well-known that functional HM deterioration is associated with progressive eyeball axial length prolongation which, in turn, entails progressive retina atrophy, pigmentary epithelium (PER) and choroids. The last cited atrophy would diminish the retina's access to the molecules that are fundamental for its functioning. Among other things, this would cause a situation of oxidative stress, as demonstrated in other retinal and macular diseases with atrophy of PER and choroids [16, 17]. Although myopia as an optical defect can be compensated by optical correction (glasses or contact lenses) or even by surgery (corneal or intraocular), the failing sight associated with HM still cannot be efficiently prevented or treated. This is basically because the causes that determine the appearance and progression of myopia are still not well-known, and it seems that a multifactor element exists. Not knowing how these factors interrelate means that myopia, particularly HM, is currently one of the main causes of blindness worldwide. All in all, myopia is a complex multifactorial problem with a worldwide prevalence and is one of the main causes of visual impairment and blindness anywhere in the world.

## 2. Factors and Molecules Involved

The factors involved in the appearance, progression and emergence of myopia-related complications can be classified into two groups: genetic and environmental/socio-cultural. Many studies have demonstrated the importance of hereditary factors [18]. Genetics have related several growth factors with myopia and HM [18, 19]. Although it depends on the population under study, there are five that stand out in HM from the rest: Transforming Growth Factor (TGF-*β*), basic Fibroblast Growth Factor (bFGF), Insulin-like Growth Factor (IGF), Vascular Endothelial Growth Factor (VEGF) and Hepatocyte Growth Factor (HGF). Briefly, the contribution of the first three focuses on eye growth control deregulation, VEGF centres on myopic choroidal neovascularisation (CNV), whereas HGF appears to not only intervene at the vascular level, but also play a neuroprotector role. The determination of some of these growth factors has already started in ocular samples of patients and animal models. The TGF-*β*  level was high and the bFGF level was low on the sclera of animals with form-deprivation myopia [20]. In the same model, an intravitreal injection of IGF caused dioptres to increase and axial length to prolong if compared to the control group [21]. VEGF has been related with the pathogenesis of CNV in patients with HM [22], which could make it a molecular target to predict the possibilities of CNV developing, or not, in HM. Despite the role of these five factors in myopia having been repeatedly associated from a genetic perspective, it has barely been studied in human ocular samples and certainly not to characterise the distinct evolution of a myopic eye versus HM. Finally, no studies have been conducted to relate these factors with its clinical manifestations and with the structural changes in the retinas of these patients.

Nevertheless, hereditary factors are far from completely responding to the myopia problem. For instance, there are vast differences in the prevalence of myopia and HM in similar populations [23]. Therefore, it is feasible to think that environmental/socio-cultural factors could answer this question. Since a few decades ago until the present-date, many studies have evidenced a relation of the excessive accommodation that close-up work, level of studies or daily reading hours with myopia demand [24, 25]. This situation could lead to constant ciliary muscle contraction, which hinders correct accommodation, to which the eye responds mistakenly with a higher eye growth rate (= myopia). Nevertheless, the attempts that have been made for centuries to prevent progressive myopia through the influence of environmental/socio-cultural factors are debatable in NM and have failed in HM [26]. For instance, the “Correction of Myopia Evaluation Trial” (COMET) stands out, which demonstrated that using progressive lenses, as opposed to simple ones, as a means to correct progression in myopia only proved effective for the first year, only by 0.2 D, and no difference was found in the next 2 years. The authors concluded that this minor correction does not guarantee their use in clinical practice [27, 28]. Complete knowledge of the molecular pathways involved in human ocular growth might be essential to be able to advance in these treatments. Other aspects to consider are, for instance, that postnatal eye growth is controlled by visual signals, the so-called emmetropisation phenomena. Data known from some studies have proposed that an active emmetropisation mechanism can play a role in postnatal eye development with axial length matching the focal plane. In normal new-born humans, axial length is shorter than in adults, so photoreceptors lie in front of the focal plane of an unaccommodated eye. Eye growth would prolong axial length and move photoreceptors to the focal plane. When a minus lens was placed during growth in animal myopia models, the focal plane shifted posteriorly and eyes elongated to match the displaced focal plane [29]. We also know that when wearing a positive lens, which causes images to be focused in front of the retina (myopic defocus), the eye reduces its ocular elongation rate and choroidal thickness increases to move the retina forward to meet the eye's focal plane. When wearing a negative lens, which causes images to be focused behind the retina (hyperopic defocus), the opposite happens [30].

Finally, attempts have been made with pharmacological agents to prevent myopia from developing, and they have been slightly more successful than the previous strategy. The use of drugs that act on muscarinic receptors, such as atropine [31] and pirenzepine [32], has managed to slow down myopia growth moderately. The end result was better for the first of the two drugs where a 3-year study managed to reduce it in almost 1 D and 0.23 mm of axial length. However, side effects were noted which have made its clinical routine use extremely difficult for the time being.

These two factors (genetic and environmental) undoubtedly intervene in the onset and progression of myopia. Nevertheless, we are still mostly unaware of the involved molecular mechanisms that lead to severe retinal deterioration among HM patients. What all this implies is that treating HM is probably the main pending issue in current Ophthalmology. Solving this problem is complicated by its masked diagnosis within retinal degeneration and by the fact that no molecular pattern exists to allow these patients to be characterised in order to make early diagnosis, predict evolution, provide adequate follow-up, and help develop and evaluate the therapeutic targets that remain unknown today. So in the last few years, work has begun to study the molecular processes involved in myopia. Firstly, these processes are relevant in the progression of this disease since local eye growth control actually exists, as seen in animal models where the ciliary or optic nerve section, or indeed both, does not hinder myopia development; however, they also intervene in the appearance of associated ocular complications [33]. Studies have been done in recent years on numerous molecules in relation to myopia and also on the aforementioned growth factors, dopamine, ZENK-glucagon, retinoic acid and retinoic acid receptors, crystallin, serotonin, melatonin, vasoactive intestinal peptide and enkephalins [34], and on the molecules related with oxidative stress, among which nitric oxide stands out. Knowledge of these biochemical and molecular aspects that accompany the clinical stages of myopia will enable us to better understand the process and to find therapeutic approach points.

## 3. Myopia and Oxidative Stress

Oxidative damage due to oxidative stress, as a result of an imbalance between free radical production and antioxidant defences, is associated with the impairment of a wide range of molecular species. Therefore, a role of oxidative stress has been postulated for many conditions, including atherosclerosis, inflammatory condition, certain cancer types and the ageing process. In this way, oxidative stress and antioxidant status have also been involved in different ocular diseases [35], like vitreoretinopathies or cataracts [36, 37], during a process where downregulation of antioxidants molecules resulting in high levels of free radicals. Earlier studies have established that exposure to oxidative stress causes the degeneration of photoreceptors and other cells of the neural retina in animal models [38]. It is well-known that free radicals are very unstable and highly reactive molecules, thus they exhibit a very good reaction capacity because they have unpaired electrons, and they are very reactive because of this instability. So they tend to reach stability by transferring or stealing electrons. The free radicals that derive from oxygen are known as reactive oxygen species (ROS) and are one of the major contributors of oxidative stress. They include superoxide anion (O_2_^•−^), hydroperoxyl radical (HO_2_) and hydroxyl radical (OH).

It is critical to maintain an adequate oxygen supply to the retina for the retinal function. Oxygen is delivered to the retina by a combination of two ways: the choroidal vascular bed, which lies closely behind the retina, and the retinal vasculature, which lies within the inner retina. So the presence of this dual circulation makes retinal oxygenation unique. The fact that the retina massively consumes oxygen, which is the highest oxygen consumption in the body [39], and the limitless light exposure that occurs in the retina, may well generate ROS. The abundant polyunsaturated fatty acids placed in the photoreceptor outer segment in the retina also make it susceptible to lipid peroxidation. Under physiological conditions, the mitochondrial genome encodes the oxidative phosphorylation system, where energy and ROS are generated [40]. However, ROS can be successfully scavenged by intrinsic antioxidant defence mechanisms. Antioxidants act as radical scavengers, peroxide decomposers, singlet oxygen quenchers, hydrogen donors, electron donors, synergists, metal-chelating agents and enzyme inhibitors. To detoxify ROS, both enzymatic and nonenzymatic antioxidants converge in the intracellular and extracellular environment [41]. Hence enzymatic antioxidants consist in copper/zinc superoxide dismutase (Cu/Zn SOD), manganese superoxide dismutase (MnSOD), catalase, and glutathione peroxidase (GPx) and nonenzymatic antioxidants, such as *α*-tocopherol (vitamin E), ascorbic acid (vitamin C), glutathione (GSH) and *β*-carotene. In fact a large amount of research has been carried out on the protective effect of antioxidants on different eye pathologies such as age-related cataracts [42], uveitis [43], glaucoma [44], age-related macular degeneration [45], and so forth.

ROS generation may alter proteins, deleterious peroxidation of lipids and DNA cleavage [46]. So damage to the retina by oxidative stress has been associated in situations of hypoxia [47, 48]. This could be one of the key aspects to explain the oxidative stress and myopia relationship since this circumstance would exist chronically in this disease. In relation to biometric modifications in the myopic eye, which increase the axial axis, retina vascularisation would diminish, and narrowness and other vascular alterations would appear [49]. According to this situation, Shih demonstrated that the higher the myopia, the lower the ocular pulse amplitude, a parameter which correlates strongly with refractive error and axial length [50]. As the ocular pulse amplitude is generated by choroidal blood flow, these results may reflect circulatory disturbance during myopia development. This relationship has also been demonstrated in myopia animal models [51]. Since retina vascularisation supplies the retina with a strong partial oxygen pressure, any vascular alteration would modify this supply and could produce transitory hypoxia situations, which would spontaneously go back to normal. Situations of this type actually imply ischaemia/reperfusion phenomena, which are known to also contribute to onset of lipid peroxidation products (LPP) through a mechanism presented later on [52]. The oxidative stress-generating hypoxic conditions of the myopic retina are very important in the retina due to its high blood flow, photic oxidative injury [53], and high polyunsaturated fatty acids content, which are one of the most frequent targets of free radicals (FR) that generate LPP involved in physiopathology, and have been proposed as markers to clinically manage many diseases, including myopia [54].

Transient global retinal ischaemia shares many similarities with transient global cerebral ischaemia [55]. Therefore, hypoxia-ischaemia results in the disequilibrium of the cellular prooxidant-antioxidant balance through ROS accumulation, known as oxidative stress, which has been involved as an important cytotoxicity mechanism.* In vitro* studies have displayed that ROS generation under hypoxic-ischaemic conditions in neurons occurs from three sources [56]. Firstly, an initial burst of ROS is generated by mitochondria. Thus the primary rise in the ROS production rate is caused by the mitochondrial respiratory chain, which also makes a smaller, but significant, contribution to ROS generation upon reperfusion. In response to oxygen and glucose deprivation, the second key ROS generation process is attributable to the activation of xanthine oxidase (XO), which follows the first burst of ROS with a substantial interruption and is linked with the time of ATP reduction. XO is an important enzymatic source of superoxide radicals in response to ischaemia/reperfusion in the retina [57], and its activation continues even at oxygen tensions, which suffice to weaken mitochondrial respiration. So, it is feasible to believe that XO has an even higher affinity to oxygen than that of cytochrome *c* oxidase. XO activation likely involves the sulfhydryl oxidation of xanthine dehydrogenase by converting the enzyme into an oxide reductase [58]. Finally, a third phase of Ca^2+^-dependent ROS generation, appreciated only upon reoxygenation after oxygen and glucose deprivation, is likely to be caused by the calcium-dependent activation of NADPH oxidase [59]. In fact endothelin-1 (ET-1) seems to increase the formation of superoxides in retinal microvascular pericytes, most probably by activating NADPH oxidase [60]. ET-1 is an extremely potent, long-acting vasoconstricting peptide expressed by retinal vascular endothelial cells that is able to increase calcium levels in pericytes to cause pericytes to contract and constrict pericyte-containing retinal microvessels [61].

One of the signs of the hypoxic theory is that in pathologic myopia, new vessels from choroids can grow into the normally avascular outer retina and subretinal space. Oxidative stress in the retinal pigmented epithelium and photoreceptors leads to higher hypoxia-inducible factor-1 (HIF-1) levels. Similarly in pathological settings, for example, when hypoxia correlates with retinal ischaemia, the imbalance between ROS production and the ability to scavenge these ROS by endogenous antioxidant systems may also be exacerbated. These conditions also result in high HIF-1 levels. HIF-1 is a heterodimeric basic helix-loop-helix structure with two subunits: HIF-1*α*  and HIF-1*β*. In hypoxic tissues, HIF-1*α*  expression increases, whereas HIF-1*β*, the aryl hydrocarbon receptor nuclear translocator, is constitutively expressed [61]. As a result, HIF-1 upregulates a number of vasoactive gene products, as well as VEGF, placental growth factor (PLGF), platelet-derived growth factor (PDGF-B), stromal-derived growth factor (SDF-1), and their receptors, and angiopoietin 2 (Angpt2). One of them, VEGF, causes vascular leakage, and brings about the development of new vessels when combined with Angpt2. VEGF, SDF-1 and PLGF recruit bone marrow-derived cells and PDGF-B recruits pericytes, and both circumstances lead to paracrine stimulation [62]. HIF-1 is important in the pathogenesis of subretinal neovascularisation (NV) because mice that lack a hypoxia response element in the VEGF promoter develop significantly less NV at Bruch's membrane rupture sites than wild-type mice [63]. Other previously cited hypoxia-regulated gene products, such as PDGF-B and SDF-1, have also been found to be implicated in choroidal NV, similarly to the situation in retinal NV [64]. Digoxin inhibits the transcriptional activity of HIF-1 [65] and strongly suppresses retinal and choroidal NV [66]. Oxidative stress increases in retinal pigmented epithelium and photoreceptors in pathological myopia, which may cause higher HIF-1 levels because mitochondrial ROS stabilise HIF-1 by reducing the activity of prolyl hydroxylases [67]. This is consistent with the observations made that oxidative stress exacerbates choroidal NV [68].

However in well-oxygenated cells, HIF-1*α*  proteins rapidly degrade, which essentially results in an undetectable HIF-1*α*  protein. Under normoxia conditions, HIF*α*  becomes hydroxylated at one of the two (or both) highly conserved prolyl residues located near the N-terminal transactivation domain by members of the prolyl hydroxylase domain (PHD) family [69]. In fact three prolyl hydroxylases, PHD1–3, which require O_2_, Fe^2+^, 2-oxoglutarate, and ascorbate for their catalytic activity, have been shown to hydroxylate HIF-1*α*  when overexpressed [70]. The hydroxylation of any of these prolyl residues of HIF*α*  generates a binding site for the von Hippel-Lindau tumour suppressor protein (pVHL), a component of an ubiquitin ligase complex. Consequently when oxygen is accessible, HIF*α*  is polyubiquitylated and subjected to proteasomal degradation. Interestingly, PHD proteins belong to the Fe(II) and 2-oxoglutarate-dependent oxygenase superfamily, whose activity depends absolutely on oxygen. Accordingly, the HIF hydroxylation rate is suppressed by hypoxia [69].

As previously mentioned, myopia (particularly HM) is closely associated with the appearance of severe eye diseases. Over the years, this has allowed the discovery of a relationship between oxidative damage and this disease. The first clear evidence for a relationship between oxidative stress and myopia probably came about through research conducted into patients with cataracts. In 1989 while studying the role of LPP in cataract development, Simonelli et al. [71] determined the malondialdehyde (MDA) level in clear and cataractous lenses of normal subjects and in cataractous lenses, and found not only that cataractous lenses contained more malondialdehyde than clear lenses, but also that the level was higher in diabetes and severe myopia than in idiopathic forms. This would induce the aggregation of soluble proteins to result in a fragmentation of the membrane structure. This idea was later confirmed since LPP was found to play a role in cataractogenesis, especially in myopic patients, and in nonmyopic patients to a lesser extent. Greater glutathione oxidation has also been found in the crystalline and vitreous humour in myopic patients [72], which suggests retinal involvement in the genesis of the human myopic cataract. In a similar study, the same group even evaluated that the MDA concentration in myopic cataractous lenses was higher than in diabetic lenses and seline ones, and unlike human diabetic cataractous lenses, the glutathione (GSH) level did not correlate negatively with the MDA concentration. This supports the hypothesis that the retinal origin of MDA is due to chronic local hypoxic conditions given choroidal thinness [73]. In line with these data, Bhatia et al. also reported a difference in the MDA level in cataract myopic lenses compared to patients with age-related cataracts [74]. It is also noteworthy that the SOD level was also lower in myopic patients than in patients with age-related cataracts. Nonetheless, no significant differences in MDA were found in plasma in both groups, but a difference was observed with the group of healthy controls. The SOD level in plasma was no different between any of the groups.

Another myopia-related eye disease is retinal detachment, where this hypoxia situation is temporarily emphasised. Our group found a close relation in myopic individuals between their dioptres and the level of LPP in the subretinal fluid of patients who had suffered retinal detachment. However, no correlation was found between the retinal detachment evolution time and LPP content in subretinal fluid, and it was ruled out that LPP production occurs merely through the peroxidation of the photoreceptor outer segments present in subretinal fluid. It was also suggested for the first time that oxidative damage played another role in the two main types of myopia described earlier because a statistically significant difference was found between patients with NM and those with HM [75]. The fact that hyaluronic acid depolymerisation, associated with vitreous liquefaction, appears during rhegmatogenous retinal detachment, which is particularly associated with myopia, and that this depolymerisation is induced by oxygen free radicals [76], once again agrees with the importance of oxidative stress in myopic patients. Arimura et al. [77] showed more recently that extracellular high-mobility group box 1 (HMGB1), a multifunctional protein present mainly in the nucleus cells that is released extracellularly by dying cells and/or activated immune cells, might be an important mediator in retinal detachment. HMGB1 would potentially act as a chemotactic factor for retinal pigment epithelium cell migration, which would lead to an ocular pathological wound-healing response. Interestingly, this study also displayed that induced oxidative stress triggered a massive release of HMGB1 from cells to cell supernatants.

A relationship between myopic hypoxia and another myopia-related disease and oxidative stress was also found quite recently: glaucoma. Zanon-Moreno et al. [78] showed that increased free radicals formation and/or reduced antioxidant protection mechanisms may play a pathogenic role in primary open-angle glaucoma. So different ways are involved in neuronal death in glaucoma, such as ischaemia (hypoxia), oxidative damage, glutamate excitotoxicity, nerve growth factor deprivation and autoimmunity, but the production of certain ROS is a step needed for neuronal death after neurotrophin deprivation. Shkrebets [79] demonstrated in HM patients its reduced antioxidant capacity in tears. Besides, the combination of a high hypoxia level and fluid content of SOD being diminished by more than 40% can be a good predictor of glaucoma in persons with rapidly progressive high-grade myopia.

**Figure 1:**
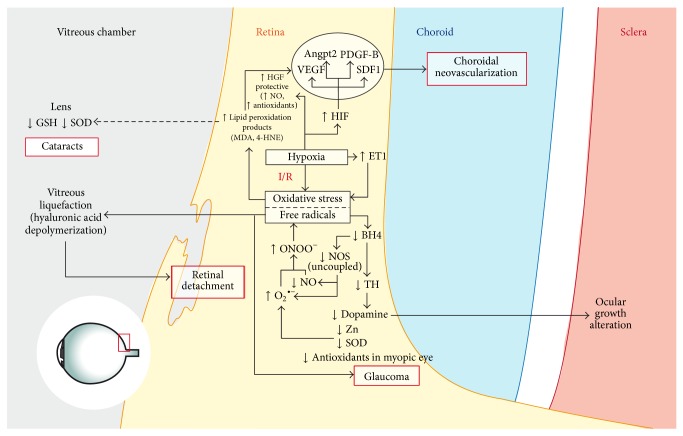
Schema explaining the main contributions of oxidative stress in myopia. 4-HNE: 4-hydroxynonenal; Angpt2: angiopoietin 2; BH4: Tetrahydrobiopterin; ET1: endothelin-1; GSH: glutathione; HGF: hepatocyte growth factor; HIF: hypoxia-inducible factor; I/R: ischemia reperfusion injury; MDA: malondialdehyde; NO: nitric oxide; NOS: nitric oxid synthase; ONOO: peroxynitrite; PDGF-B: platelet-derived growth factor beta; SDF1: stromal-derived growth factor 1; SOD: superoxide dismutase; TH: tyrosine hydroxylase; VEGF: vascular endothelial growth factor.

Not only has the relationship of oxidative stress and myopia been evidenced in associated eye diseases, but also has its development (Figure 1). Zinc is an essential catalytic, structural cofactor for numerous enzymes and other proteins. While Zn^2+^ is not redox-active under physiological conditions, it is known that lack of zinc increases oxidative stress and, accordingly, also enhances oxidative damage to DNA, proteins and lipids [80]. Apart from its role as a neuromodulator, Zn is capable of reducing oxidative stress by several mechanisms: induction of some other antioxidant proteins, molecules and enzymes, such as metallothioneins, GSH, catalase, and SOD; protection of protein sulfhydryls from oxidation by binding with them, or reduction of ^•^OH formation by competing with iron and copper ions to displace these redox active metals, which catalyse ^•^OH production from H_2_O_2_. Zinc also reduces the activities of oxidant-promoting enzymes, such as inducible nitric oxide synthase (iNOS) and NADPH oxidase, and inhibits the generation of lipid peroxidation products. In fact the eye, especially the retina and the underlying retinal pigment epithelium/choroid complex, contains high zinc concentrations. Hence several eye disorders are associated with altered zinc balance, and zinc supplementation has become a choice treatment for diseases like age-related macular degeneration [81].

In a myopia animal model, Huibi et al. [82] discovered how reduced SOD, nitric oxide synthase (NOS) activity and nitric oxide (NO) content existed in the retinal pigmental epithelium choroid homogenate, and also how the administration of trace element zinc was able to not only increase these three parameters in myopic chick eyes, but also inhibit the elongation of axis oculi and increased dioptres. The quantity of zinc in the retina of myopic animals also diminished if compared to a normal eye. Logically, these levels recovered in the treated group, which indicates certain metabolic disorders in the connective tissue system, first of all in the scleral membrane and then in the antioxidant defence system [83], in parallel to myopia prevention [82, 84]. In line with this idea, a high level of zinc in serum may be found in HM patients with retinal detachment, although the level of subretinal fluid can be low. In other words, an inversely proportional relationship exists between zinc in serum and zinc in subretinal fluid [85, 86]. Apart from being found in this fluid, altered zinc quantities have been encountered in tears, scalp hair and myopic people. Interestingly, a recent whole exome sequencing study identified a causative gene in a Chinese family with autosomal dominant high myopia, and replicated their results in a sporadic cohort [87]. Afterwards, Tran-Viet et al. [88] performed mutation screening in a US cohort for zinc finger protein 644 gene (*ZNF644*) and recognised a new missense mutation, which supports the notion that* ZNF644* may be a causative gene for HM. The protein encoded by this gene is a zinc finger transcription factor, which may play a role in eye development [87], which is a small protein characterised by the coordination of one zinc ion, or more, to stabilise the fold. ZNF644 functions as a transcriptional factor and is ubiquitously expressed in several tissues, such as the eye, liver and placenta. However, the biologic function and mechanism of this gene in HM pathogenesis are still unclear [89, 90].

As a final point, anthocyanins, water-soluble glycosides of polyhydroxyl and polymethoxyl derivatives of 2-phenylbenzopyrylium or flavylium salts, have shown to be potent antioxidants, superior to other well-known antioxidants such as alpha-tocopherol or 6-hydroxy-2,5,7,8-tetramethychromane-2-carboxylic acid (Trolox) [91]. Berry anthocyanins seem to help vision in several ways as well as by increasing circulation within the retina capillaries, improving night vision by enhanced generation of retinal pigments, decreasing molecular degeneration and diabetic retinopathy and improving or preventing glaucoma, retinitis pigmentosa, myopia and cataracts [92, 93]. However, the studies on the effects of anthocyanins on vision are still contradictory. So, more extensive research is necessary to further sustenance and validates the data to support the described ocular health benefits of anthocyanins.

## 4. Nitric Oxide as a Key Element

First of all, NO plays a relevant role in the eye as a neuromodulator and vasodilator, but it also acts as both a regulator of eye growth and a smooth muscle relaxant, which are especially and evidently interesting for myopia [94]. NO is an important signaling molecule with multiple pivotal roles in the neural and cardiovascular systems, as well as in inflammatory response. Due to its unpaired electron, NO is a free uncharged radical, with the unpaired electron being closer to the nitrogen atom of the NO molecule: N^•^=O [95].

Both nitric oxide (NO) underproduction and overproduction can lead to various eye diseases, so the interest in using NO donors and inhibitors to treat or prevent these eye diseases, including myopia [96], has increased in recent years. Firstly, attempts have been made to study its role in the onset and development of myopia by using inhibitors of NO and determining the activities of several NOS isoforms. An intravitreal injection of N-omega-nitro-L-arginine methyl ester (L-NAME; an inhibitor of NOS) inhibits myopia development in the two most widely used animal myopia models: form deprivation myopia (FDM) and lens-induced myopia (LIM). This suggests that NO modulates a common retinal pathway, which leads to LIM and FDM [97, 98]. In similar studies, it has been demonstrated that the use of L-NAME has effects not only on choroids, but also on scleral proteoglycan synthesis [99]. Fang et al. [100] did a time course of retinal NOS activity and cyclic GMP (cGMP) concentration using an FDM model in guinea pigs. NO can activate soluble guanylate cyclase, and thereby increases cyclic GMP (cGMP) levels. This, in turn, activates cGMP-dependent protein kinases to cause physiological or pathological effects on target protein phosphorylation [101]. Retinal NOS activity in FDM groups was lower than in controls after 7 days of FD and was higher than in controls after 14 and 21 days of FDM. cGMP concentration exhibited a similar increase to NOS activity in sustained FDM, which suggests that the function of strong NOS activity may be mediated by cGMP, at least in part. It has been previously suggested that the retinal degenerative lesion in HM is caused by iNOS overproduction [96].

The existence of three NOS isoforms, inducible (iNOS), neuronal (nNOS) and endothelial (eNOS), in the eye with very different functions [94] complicates the interpretation of this result. In order to elucidate this point, iNOS expression in retina-RPE-choroid lowered in a chick FDM model after 7 days, but the expression of nNOS and eNOS was the same as in the controls and was found mainly in the outer part of the photoreceptor layer, and in outer and inner RPE and choroid parts (external parts of the retina), but only nNOS was present on the outer nuclear layer. The swift myopia development in chick FDM and the short period used (7 days) did not confirm whether iNOS is responsible for retinal damage in consolidated myopia. Nevertheless, the authors reported a predominant expression of iNOS, but not of nNOS and eNOS, which indicates the tissue-specific regulation of the iNOS gene. This confirms that the three NOS isoforms play different roles in the regulatory mechanisms of a myopic eye; including eye growth regulation [102]. The most recent usage attributed to the specific inhibitors of each isoform is now beginning to elucidate the importance of each one in myopia development [103].

The important role of NO in myopia has also been justified for its interrelation with other molecules implied in myopia, such as retinoic acid [99], melatonin [104] and serotonin [105], and its relationship with dopamine is particularly important [106].

The main acknowledged dopamine functions are light adaptation and retinal circadian rhythm regulation. A rise in retinal dopamine levels stimulates dopaminergic receptors D1 and D2, which are present throughout the retina, and triggers a signal that inhibits axial growth once the eye has reached emmetropisation [107]. Dopamine and NO, which are released in the retina under light-adaptation conditions, appear to be critical for myopia prevention and light adaptation, and also for uncoupling the gap junctions between retinal cells, which may play a key role in ocular growth regulation through some DA and NO actions [108]. Therefore, along with NO, dopamine is one of the retinal neurotransmitters involved in the signalling cascade that controls eye growth [106]. Despite some evidence supporting that dopamine acts upstream of NO [109], the relationship between both could be more complex given the crosstalk between different pathways. It has been demonstrated that the retinal dopamine level is lower in different myopia animal models, is accompanied by a low dopamine biosynthesis rate and is associated with reduced tyrosine hydroxylase activity (TH), this being the rate-limiting enzyme in dopamine biosynthesis [110]. If we take into account that both dopamine and NO are light-activated neuromodulators, and that both are released by amacrine cells [111, 112], we wondered whether NO had something to do with the reduction of dopamine in myopic eyes. Indeed Ara et al. [113] described how the presence of peroxynitrite inactivates TH in dopaminergic neurons in culture, which has been prevented in mice overexpressing copper/zinc SOD. It has been suggested how in the face of an oxidative stress situation, deficiency by tetrahydrobiopterin (BH4) oxidation would occur, where BH4 is a critical cofactor required for the synthesis of both catecholamines and NO, which would “uncouple” NOS to produce less NO and more superoxide radical and which, in turn, could contribute to BH4 oxidation (even through peroxynitrite formation) to close the positive feedback circuit. This further exacerbates ROS-mediated oxidative damage and apoptosis, a mechanism that has already been suggested in some neurodegenerative diseases [114, 115]. Heller discovered how the presence of antioxidants in endothelial cells is necessary to protect BH4 from its oxidation and to allow correct NO synthesis, which supports this mechanism [116].

As mentioned in the second section, genetics has constantly shown that myopia is related with various growth factors, and some are interesting for the object of this review. For instance, 4-hydroxynonenal, a strong active lipid peroxidation product, increases VEGF expression in human retinal pigment epithelial (RPE) cells due to an associated increase in intracellular oxidative stress [117]. It is also noteworthy how bFGF is released by Müller glial cells in the ischaemic-hypoxic retina, which induces secretion of VEGF and HGF [118]. Yet of them all, the latter clearly stands out. Therefore, the known genetic relation between growth factors and myopia could become a molecular pathway where these growth factors could help explain the physiopathological mechanisms of myopia given its strong interaction with the aforementioned oxidative stress elements. As previously mentioned, HGF plays not only a vascular role, but also a neuroprotector one [119, 120]. HGF expression is upregulated under hypoxic conditions, increases retinal vascular permeability [121, 122] and is also capable of increasing eNOS activity and NO production [123]. It forms part of the eNOS signalling pathway, a contributor to vasodilation in microvascular territories and determines the calibre [124]. Interestingly, a genetic variation in HGF has been found among Chinese children, which has been associated with an altered retinal vessel diameter, a parameter related to the pathogenesis of microvascular disease [125].

HGF has also been demonstrated to be capable of protecting the antioxidant system: it is able to raise hepatic GSH levels by increasing gamma-glutamylcysteine synthetase (gamma-GCS) activity, the rate-limiting enzyme of GSH biosynthesis [126]. It produces the recovery of mitochondrial GSH and bcl-2 and is capable of protecting human RPE from apoptosis induced by glutathione depletion [127]. In a ceramide-induced apoptosis model in RPE cells, it has been reported to activate antioxidant genes, such as catalase [128], and is capable of having a strong neuroprotective effect on photoreceptor cells in a retinal detachment model [129]. In this way, HGF can become a transcendental molecule in the mechanisms against myopic hypoxia and in preventing oxidative damage and is thus of intense therapeutic interest.

## 5. Conclusion and Perspectives

Thus there is evidence that oxidative stress forms part of the molecular bases that participate in the growth and appearance of diseases associated with myopia, such as a portion of the underlying mechanisms associated with the previously shown genetic and environmental factors. Over the next few years, we believe that research should advance in two directions. On the one hand, conduct a systematic and complete study of the changes produced in both valid animal models to study human myopia and in patients with myopia. This study could centre on three groups of substances: neurotransmitters (such as dopamine and acetylcholine), growth factors (like HGF and VEGF) and oxidative stress-related parameters (paying special attention to NO and lipid peroxidation products) which help construct the physiopathological molecular mechanisms present in the disease. On the other hand, relate these pathways with the clinical manifestations and structural changes present in myopia, which help improve both today's differential diagnosis between NM and HM and their treatment, which would help curb the foreseen increase in myopia worldwide.
